# Berberine and rifaximin effects on small intestinal bacterial overgrowth: Study protocol for an investigator-initiated, double-arm, open-label, randomized clinical trial (BRIEF-SIBO study)

**DOI:** 10.3389/fphar.2023.1121435

**Published:** 2023-02-15

**Authors:** Huaizhu Guo, Siqi Lu, Jindong Zhang, Chen Chen, Yanlin Du, Kun Wang, Liping Duan

**Affiliations:** ^1^ Department of Gastroenterology, Peking University Third Hospital, Beijing, China; ^2^ International Institute of Population Health, Peking University Health Science Center, Beijing, China

**Keywords:** small intestinal bacteria overgrowth (SIBO), berberine (BBR), rifaximin, microbiota, breath test

## Abstract

**Introduction:** Small intestinal bacterial overgrowth (SIBO) leads to non-specific abdominal discomfort and nutrient malabsorption. Currently, rifaximin is widely applied in SIBO based on its antibacterial and non-absorbable nature. Berberine is a natural component of many popular medicine plants that ameliorates intestinal inflammation in humans through its modification of the gut microbiota. Potential effect of berberine to the gut may provide therapeutic target for SIBO. We aimed to evaluate the effect of berberine compared with rifaximin on SIBO patients.

**Methods:** This is an investigator-initiated, single-center, open-label, double-arm randomized controlled trial, termed BRIEF-SIBO (Berberine and rifaximin effects for small intestinal bacterial overgrowth). In total, 180 patients will be recruited and allocated to an intervention group (berberine) and a control group (rifaximin). Each participant will receive one 400 mg drug twice a day (800 mg daily) for 2 weeks. The total follow-up period is 6 weeks from the start of medication. The primary outcome is a negative breath test. The secondary outcomes include abdominal symptom relief and alteration in gut microbiota. Efficacy assessment will be performed every 2 weeks, as well as safety assessment during the treatment. The primary hypothesis is that berberine is not inferior to rifaximin for SIBO.

**Discussion:** The BRIEF-SIBO study is the first clinical trial assessing the eradication effects of 2 weeks of berberine treatment in SIBO patients. The effect of berberine will be fully verified by using rifaximin as the positive control. The findings of this study may have implications for the management of SIBO, especially increasing the awareness of both physicians and patients who are suffering from long-term abdominal discomfort and avoiding excessive examination.

## 1 Introduction

Small intestinal bacterial overgrowth (SIBO) is defined as the presence of an abnormally excessive amount of bacterial colonization in the small bowel (Quigley et al., 2020; Bushyhead and Quigley, 2022). Primary or secondary motility abnormalities destroy the ability of the small intestine to prevent colon bacterial translocation (Bohm et al., 2013). Meanwhile, ileocecal valve dysfunction leads to colonic bacterial regurgitation (Miller et al., 2012). It is believed that symptoms linked to SIBO consist of bloating, diarrhea and abdominal pain/discomfort. Steatorrhea, vitamin B_12_ deficiency and malnutrition can be seen in more severe cases.

The gold standard for the diagnosis of SIBO is quantitative culture of small intestine aspirates. The American Gastroenterology Association (AGA) recently recommended a new threshold at >10^3^ colony-forming units per milliliter (CFU/mL) on fresh aspirate culture instead of >10^5^ CFU/mL based on a large-scale study (Leite et al., 2019), derived from subjects with altered intestinal anatomy because the bacterial level in normal subjects rarely exceeds 10^2^ CFU/mL (Pimentel et al., 2020). An alternative method is the measurement of exhaled hydrogen and methane gas, which is considered a non-invasive, safe, useful, and cost-efficient test (Hammer et al., 2022). The North American Consensus recommended that a rise in hydrogen of ≥20 part per million (ppm) or methane levels ≥10 ppm by 90 min during glucose or lactulose breath test was considered positive (Rezaie et al., 2017).

SIBO is now commonly diagnosed and closely associated with many gastrointestinal diseases, such as irritable bowel syndrome (IBS) (Ghoshal et al., 2020), inflammatory bowel disease (IBD) (Shah et al., 2019), pancreatitis (El Kurdi et al., 2019), non-alcoholic liver disease (Wijarnpreecha et al., 2020), colorectal cancer and abdominal surgery. Based on the similarity of the clinical manifestation profiles between IBS and SIBO, the prevalence of SIBO in IBS patients has been widely reported to range from 4% to 84%, with an overall pooled prevalence rate of 38% (Chen et al., 2018). A meta-analysis of case‒control studies found that SIBO prevalence in patients with IBS was 35.5% (95% CI 33.6–37.4) and 29.7% (95% CI 27.6–31.8) in controls based on breath tests, while culture-based studies yielded a SIBO prevalence of 33.5% (95% CI 30.1–36.9) in patients with IBS and 8.2% (95% CI 6.8–9.6) in controls with a cutoff value of 10^3^ CFU/mL (Shah et al., 2020). The diagnostic modality actually influences the prevalence of SIBO in consideration of the sensitivity and specificity of breath tests.

Currently, rifaximin is widely applied considering its broad-spectrum and non-absorbed nature to achieve low gastrointestinal absorption while retaining good antibacterial activity, which is thought to be effective and safe for the treatment of SIBO (Gatta and Scarpignato, 2017). Studies have shown significant symptom remission of rifaximin therapy in IBS patients with SIBO (Moraru et al., 2014; Liu et al., 2016; Leite et al., 2018; Rezaie et al., 2019; Tuteja et al., 2019). Unfortunately, no responsiveness and recurrence after eradication of SIBO limit antibiotic usage. Rezaie et al. (2019) reported that 48/93 (51.6%) were non-responders after 2 weeks of low-dose rifaximin treatment. For those responders, the average time of recurrence was 94.86 ± 38.6 days, and 38 (84.4%) of the 45 patients experienced symptom recurrence by the end of the 18-week observation phase. Moreover, we must take into account that rotating antibiotic regimens lead to drug resistance and risks of *Clostridium difficile* infection. Medication for SIBO remains confusing and limited.

Berberine is a natural pentacyclic isoquinoline alkaloid extracted from many popular medicine plants such as the genus *Berberis*, *Coptis* and *Hydrastis* (Wang et al., 2017). There is already evidence that the structural and numerical changes in the gut microbiota under pathological conditions can be reversed by berberine (Jia et al., 2019), which mediates modulatory effects on microglial activation and visceral hypersensitivity (Zhang et al., 2021), and ameliorates intestinal inflammation in humans through antibacterial action (Gong et al., 2017; Habtemariam, 2020). Berberine enhanced the composition of beneficial bacteria such as *Bacteroides*, *Bifidobacterium*, *Lactobacillus*, and *Akkermansia* (Fang et al., 2021). Furthermore, it could alleviate colon inflammation by regulating interferon-gamma and interleukin-17A-producing lamina propria CD4 (+) T cells (Takahara et al., 2019). It has been proven to significantly decrease diarrhea and abdominal pain scores in diarrhea-predominant IBS patients (Chen et al., 2015). Berberine effects to the gut may provide therapeutic targets for SIBO. Chedid et al. (2014) confirmed that herbal therapy containing berberine is equivalent to rifaximin for the resolution of SIBO. However, there is little evidence investigating the potential effect of berberine as a single agent in SIBO patients. In the present study, we aimed to evaluate the effects of berberine and rifaximin on patients with SIBO (BRIEF-SIBO study). The effectiveness outcomes included a negative lactulose hydrogen methane breath test (LHMBT) and clinical symptom remission. Different gut microbiome spectra in SIBO patients treated with berberine and rifaximin may provide a potential explanation.

## 2 Methods and analysis

### 2.1 Trial design

This is an investigator-initiated, single-center, open-label, two-arm randomized controlled trial from Peking University Third Hospital, Beijing, China, termed BRIEF-SIBO. The study aims to evaluate the efficacy and safety of berberine for SIBO patients compared with rifaximin as the positive control. This study was registered on the Chinese Clinical Trial Registry platform under number ChiCTR2200057554. The study protocol, informed consent and other documents were reviewed and approved by the Peking University Third Hospital Medical Science Research Ethics Committee under number 2022-021-02. This protocol complies with the Standard Protocol Items: Recommendations for Interventional Trials (SPIRIT) (Chan et al., 2013).

### 2.2 Patient recruitment and eligibility

Patients who have at least one of the major symptoms, including abdominal pain, distension, diarrhea or constipation, with positive LHMBT will be enrolled in this study. The inclusion criteria were as follows: 1) Aged ≥18 and ≤65 years old; 2) Complained of abdominal pain, distension, constipation or diarrhea for over 6 months, and at least one of them up to a moderate or severe degree according to the gastrointestinal symptom rating scale (GSRS); 3) Positive hydrogen breath test with elevated or normal methane gas; 4) Voluntarily joined the study and completed the case report form, LHMBT, colonoscopy and blood biochemical examination; and 5) Took drugs according to the required rules with good compliance.

Patients who were found to have gastrointestinal organic diseases detected by endoscopy or digestive tract surgery history were excluded. Patients with severe heart, liver, lung, kidney, blood, endocrine, and nervous system diseases, or severe respiratory tract, digestive tract, urinary tract infections or mental disorders will temporarily stop entering treatment until professional clinicians access their conditions. Patients who were taking antibiotics and acid suppression drugs for more than 3 days during the past month or probiotics, laxatives, antidiarrheal or prokinetic agents within 2 weeks will not be eligible for participation. Pregnant or lactating women will also be excluded. Patients who firmly report rifaximin or rifamycin allergy will not participate, as it is possible to be randomized to the rifaximin group. All patients will be identified through gastroenterologists for their patients with non-specific abdominal symptoms. Two principal researchers of the project team are responsible for explaining the background, purpose, process, risks and benefits of this study and obtaining informed consent.

### 2.3 Visit and procedure

#### 2.3.1 Screening

**FIGURE 1:**
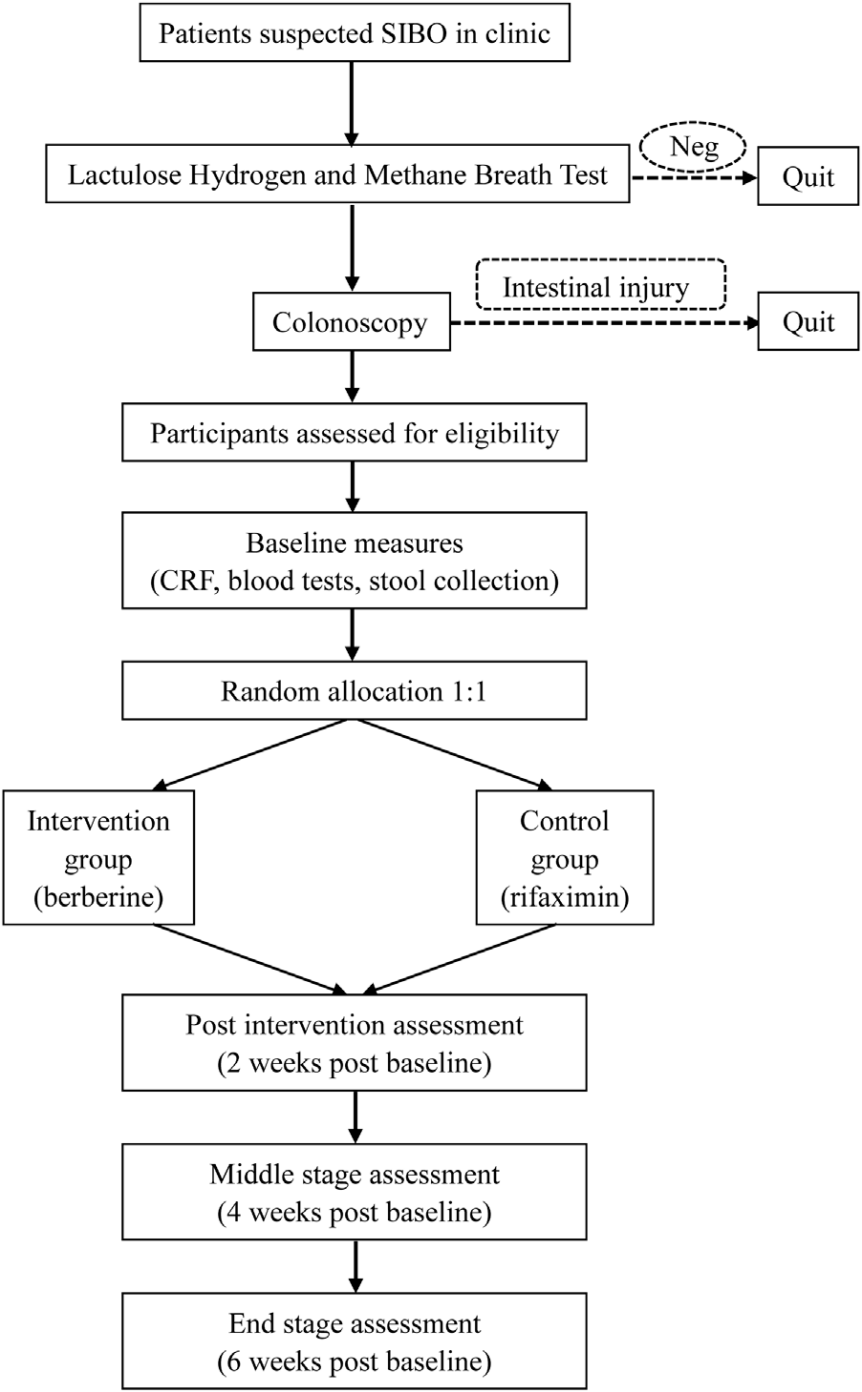
Flow diagram based on the Consolidated Standards of Reporting Trials guidelines (CONSORT): Neg, negative; CRF, case report form.

The study flow diagram is shown in Figure 1 (Schulz et al., 2010). We will first perform LHMBT for those suspected to have SIBO and exclude negative patients. The next examination is colonoscopy to ensure that they do not have organic colon diseases. Baseline measures include a case report form (shown in Supplementary Material), regular blood tests, stool collection for calprotein and 16S rRNA sequencing. Blood samples will be collected and shipped to the core laboratory. Stool samples will be stored at −80°C.

Before LHMBT, all subjects are supposed to refrain from antibiotic use and discontinue probiotics, laxatives, antidiarrheal and prokinetic agents for 2 weeks. To minimize basal hydrogen excretion, dietary restriction and avoidance of smoking for at least 24 h prior to the test and during the test are recommended. Furthermore, patients are required to avoid coarse grains, milk, juice, and alcohol in the evening before the test. Fasting for 8–12 h before the procedure is needed. Before the examination, subjects use 20 mL antiseptic mouthwash (0.05% chlorhexidine) to eliminate fermentation by oral bacteria. End-expiratory breath samples will be collected just before the ingestion of 10 g (15 mL) of lactulose in a 250 mL water solution. Gas samples are collected every 15 min until 90 min and tested immediately by the methane-hydrogen breathing analyzer DA6000 (Sunvou Medical Electronics Co., Ltd., Wuxi, China). A rise in hydrogen of ≥20 part per million (ppm) by 90 min with elevated or normal methane was considered positive. Patients with only methane levels ≥10 ppm are not eligible (Rezaie et al., 2017).

#### 2.3.2 Intervention

In total, 180 patients will be recruited and divided into two groups: the berberine group (Berberine Hydrochloride Tablets, H51022193, Chengdu Jinhua Pharmaceutical Co., Ltd., China) and the rifaximin group (Xifaxan, H20181212, Alfasigma S.p.A, Italy). Each participant will receive only one 400 mg drug twice a day (800 mg daily) for 2 weeks. The recommended dose of berberine is mainly determined by the advice of pharmacological experts, while that of rifaximin is based on past clinical trial conclusions. The first participant was enrolled on 15 March, 2022, and the study is expected to be terminated by July 2023 with the anticipated inclusion of 180 participants.

#### 2.3.3 Follow-up

The total follow-up period is 6 weeks from the start of medication. Efficacy assessment will be performed every 2 weeks using GSRS (Dimenäs et al., 1993), Bristol Stool Form (BSF) and breath tests, as well as safety assessment during the first 2 weeks of treatment. Each specific visit and measurement are summarized in Table 1. Multiple methods, such as telephone and online follow-up, are carried out to improve patient compliance if room consultation is impossible. Participants will return unused tablets and report reasons for non-compliance at the first visit (Kini and Ho, 2018). If a patient decides to withdraw, the follow-up will be stopped.

**TABLE 1:** Standard protocol items: Adapted recommendations for interventional trials (SPIRIT) schedule: Enrollment, interventions, and follow-up.

	Screening	1 week	2 weeks	4 weeks	6 weeks
Case report form	×				
GSRS and BSF	×		×	×	×
Breath test	×		×	×	×
Blood test	×				
Enteroscopy	×				
Stool 16S rRNA sequencing	×		×	×	×
Medication record		×	×		
Adverse event		×	×		

Legend: GSRS, gastrointestinal symptom rating scale; BSF, bristol stool form.

### 2.4 Results

#### 2.4.1 Effectiveness outcomes

The primary outcome is a negative LHMBT. It is defined as a rise of hydrogen less than 20 ppm and methane less than 10 ppm within 90 min compared with screening tests.

The secondary outcomes include abdominal symptom relief and alterations in gut microbiota. The key secondary outcome we designed was similar to the relief of IBS global symptoms (Pimentel et al., 2011). Abdominal symptom remission is required to meet at least one of the following criteria: 1) The pain or distension scores of GSRS decrease at least 30% from baseline; 2) Hard stool (1/2 in BSF) and loose stool (6/7 in BSF) reduce more than 50% from baseline.

#### 2.4.2 Assessment of harms

The study investigator will exclude those with any physical problems that may limit participation with berberine or rifaximin exposures. Participants will be asked to report any adverse events (AEs) experienced during the study period. Participants with adverse events (e.g., dizziness, headache, loss of taste, dull sensation, diplopia, vertigo, palpitations, hot flashes, dyspnea, hematochezia, rash, back pain, muscle spasm, muscle weakness, hematuria, fever, flu-like symptoms after intervention) will be advised to consult their physician to provide healthcare as appropriate. The investigator will record the details of the frequency and severity of adverse events.

### 2.5 Randomization and allocation

Participants will be randomized after baseline assessment to either berberine or rifaximin intervention with a 1:1 allocation. A laboratory staff member, not the investigator, used Statistical Package for Social Science Version 26.0 software (SPSS V.26.0) to produce 180 random numbers after setting the starting point according to the simple randomization. The patient codes were divided into two groups (A for berberine and B for rifaximin) according to the size of the number to form a random allocation table. The corresponding information of each code was assembled into the envelope seal. Thus, the principal investigator will remain blinded to the randomization process. Participants will be aware of the group to which they are allocated.

### 2.6 Sample size

We calculated the sample size based on the primary outcome of eradication rate. Gatta and Scarpignato (2017) published a meta-analysis in which the overall eradication rate of rifaximin for SIBO patients according to per-protocol analysis was 72.9%. Therefore, the null hypothesis is postulated to be a rate of 72.9% (*P*_*0*_). We proposed that the effective rate of berberine is 69.8% (*P*_*1*_) (Wang et al., 2002; Chen et al., 2015). Using the non-inferiority design with unilateral *α* = 0.025 and power 1-β = 0.8, 82 participants will be enrolled in each group. However, assuming a 10% attrition rate, we will aim to recruit 90 participants per group.

### 2.7 Criteria for discontinuing or modifying allocated interventions

If a severe or unanticipated adverse event that may influence the risk-benefit ratio of the study occurs, the principal investigator must report to the ethics committee and permanently discontinue the treatment. Peking University Third Hospital, the sponsor of this study, will pay for medical expenses and provide economic compensation for the injuries related to this study. Study treatment must be stopped permanently in the event of unintended pregnancies. In addition, participants can withdraw from the trial at any stage for any reason. For patients with limited symptom response, we will provide alternative interventions including switching medication, probiotics and dietary guidance.

### 2.8 Data management

The original data will be correctly, completely and clearly recorded in the written case report form and observation questionnaire. After being reviewed and signed by the principal investigator, the records should be sent in a timely manner to the clinical research data manager. The Microsoft Excel database will be used by two-person and two-machine input for checking. Data review will be performed regularly by the study monitoring committee to ensure completeness and accuracy. The monitor will finish a data inspection report for each participant, including the study completion, inclusion and exclusion criteria, pharmaceutical compliance, concomitant medication, adverse events, *etc.* During this period, the investigator should be required to answer if any problem is found, and the monitor will be informed in time. The exchange of questions and answers between them should be sent in the form of a question sheet for future reference.

The losses and dropouts will be carefully recorded to ensure study reliability. The next participant who meets the eligibility criteria will replace the last participant who withdraws from the research until reaching the calculated sample size. All participant information will be stored in a safe place anonymously by the responsible researcher as required by Chinese relevant laws to guarantee the security of privacy. The study results will be disseminated in a peer-reviewed journal and at conferences without any privacy information of participants.

### 2.9 Statistical analysis

Patients who finish at least one postmedication evaluation after randomization and subsequent treatment constitute the study full analysis set (FAS) as well as the primary group of efficacy assessment, according to the intention to treatment (ITT) principle. Patients who meet the criteria and complete the observation before medication without other affected treatments during the trial will be included in the study per protocol set (PPS) as the secondary group of efficacy assessment. The study safety set consists of all cases who have safety evaluation data after taking at least one kind of drug. Rubin’s method of multiple imputation will be performed for missing values to restore natural variability and uncertainty. The complete and imputed datasets will be combined and analyzed (Little et al., 2012). Complete case analysis is used for fecal microbiota on the nature of the outcome.

Analysis will be conducted using SPSS V.26.0. The quantitative and qualitative variables were reported as mean ± standard deviation (SD), median ± interquartile range (IQR), and number (frequency). Univariate analysis of variance (ANOVA) will examine differences between groups for variables with continuous data. χ2 tests will examine differences between groups for categorical variables. Unadjusted ANOVA and adjusted analysis of covariance models will compare differences in scores from baseline and postintervention data within and between groups using FAS and PPS. Variable correlations will be analyzed through Spearman’s correlation analysis. Statistical significance was defined as a *p*-value less than 0.05.

## 3 Discussion

The pharmacology of berberine has been extensively explored and revealed multifunctional activities, including anti-inflammatory (Wang et al., 2017), anticancer (Chen et al., 2019; Sun et al., 2019; Gong et al., 2020), antidiabetic (Gong et al., 2017), antihyperlipidemic and cardioprotective effects, based on its regulation of gut microbiota. To the best of our knowledge, the BRIEF-SIBO study is the first clinical trial assessing the eradication effects of a two-week intervention with berberine in SIBO patients. The effect of berberine will be fully verified by using rifaximin as the positive control. The findings of this study may have implications for the management of SIBO, especially increasing the awareness of both clinicians and patients who suffer from long-term abdominal discomfort and avoiding excessive examination. We also expect to promote clinical diagnosis and efficient management. Intervention patterns will refine and inform the development of future berberine medication designed for patients with SIBO.

The goal of therapy in SIBO is not only to eradicate the small intestinal microbiota but also to improve symptoms (Quigley et al., 2020). Thus, we set abdominal symptom remission as an important point of effectiveness. The secondary outcomes also include other symptom improvements from GSRS and alterations in the gut microbiome. Different microbiome spectra in SIBO patients after treatment with berberine and rifaximin may provide a potential explanation. Predictors may be considered in clinical practice to target SIBO patients most likely to benefit from the varied intervention.

## 4 Trial status

This study was prospectively registered on the Chinese Clinical Trial Registry platform under number ChiCTR2200057554 (15 March, 2022). The study is ongoing and has not completed participant recruitment at the time of submission. Recruitment is expected to be completed by July 2023.
