# Supplementary material for: Berberine and rifaximin effects on small intestinal bacterial overgrowth: Study protocol for an investigator-initiated, double-arm, open-label, randomized clinical trial (BRIEF-SIBO study)
Source: Front Pharmacol. 2023 Feb 15;14:1121435. doi: 10.3389/fphar.2023.1121435 (PMC9974661; doi:10.3389/fphar.2023.1121435)
Supplement: Supplementary file 1 [file Table1.DOCX]

**Supplementary Material**

**Case Report Form**

| **PART A** General information  Gender：□male=0 □female=1 Birth：year□□□□ month□□ day□□  Height： □□□cm Weight： □□□kg  Smoking： □no=0 □yes=1 year□□ frequency□□  Alcohol： □no=0 □yes=1 year□□ frequency□□  History：  □Cardiovascular disease: ___________  □Pulmonary disease: ___________  □Digestive system disease: ___________  □Cancer: ___________  □Endocrine disease: ___________  □Haematological disease: ___________  □Autoimmune deficiency: ___________  □Kidney disease: ___________  □Food or drug allergy:  □Sensitivity disease: ___________  □Others: ___________  **PART B** Lactulose hydrogen and methane breath test □positive □negative   \| Time (min) \| H_2_ (ppm) \| CH_4_ (ppm) \| CO_2_ (%) \| O_2_ (%) \| \| --- \| --- \| --- \| --- \| --- \| \| 0 \|  \|  \|  \|  \| \| 15 \|  \|  \|  \|  \| \| 30 \|  \|  \|  \|  \| \| 45 \|  \|  \|  \|  \| \| 60 \|  \|  \|  \|  \| \| 75 \|  \|  \|  \|  \| \| 90 \|  \|  \|  \|  \|   **PART C** Symptom score  **C1**: Gastrointestinal Symptom Rating Scale (GSRS)   \| Severity of discomfort \| none \| little \| light \| moderate \| moderate-heavy \| heavy \| severe \| frequency \| \| --- \| --- \| --- \| --- \| --- \| --- \| --- \| --- \| --- \| \| 1.pain \|  \|  \|  \|  \|  \|  \|  \|  \| \| 2.heartburn \|  \|  \|  \|  \|  \|  \|  \|  \| \| 3.reflux \|  \|  \|  \|  \|  \|  \|  \|  \| \| 4.throat discomfort \|  \|  \|  \|  \|  \|  \|  \|  \| \| 5.nausea \|  \|  \|  \|  \|  \|  \|  \|  \| \| 6.bowel sound \|  \|  \|  \|  \|  \|  \|  \|  \| \| 7.belch \|  \|  \|  \|  \|  \|  \|  \|  \| \| 8.distension \|  \|  \|  \|  \|  \|  \|  \|  \| \| 9.gas \|  \|  \|  \|  \|  \|  \|  \|  \| \| 10.diarrhea \|  \|  \|  \|  \|  \|  \|  \|  \| \| 11.constipation \|  \|  \|  \|  \|  \|  \|  \|  \| \| 12.loose stool \|  \|  \|  \|  \|  \|  \|  \|  \| \| 13.hard stool \|  \|  \|  \|  \|  \|  \|  \|  \| \| 14.urgent stool \|  \|  \|  \|  \|  \|  \|  \|  \| \| 15. tenesmus \|  \|  \|  \|  \|  \|  \|  \|  \|   **C2**: Bristol stool form (the sum of percentage is 100)   \| 1.□no=0 \| □yes=1 \| percentage： % \| \| --- \| --- \| --- \| \| 2.□no=0 \| □yes=1 \| percentage： % \| \| 3.□no=0 \| □yes=1 \| percentage： % \| \| 4.□no=0 \| □yes=1 \| percentage： % \| \| 5.□no=0 \| □yes=1 \| percentage： % \| \| 6.□no=0 \| □yes=1 \| percentage： % \| \| 7.□no=0 \| □yes=1 \| percentage： % \|     **C3**: IBS-Symptom severity scoring system Score:  1.Do you currently suffer from abdominal pain? □no □yes  If yes, how severe is your abdominal pain?  0%  No pain  50%  Quite severe  100%  Very severe  Please enter the number of days that you get the pain in every 10 days: /10  2. Do you currently suffer from abdominal distension? □no □yes  If yes, how severe is your abdominal distension?  (*women, please ignore distension related to your periods)  0%  No distension  50%  Quite severe  100%  Very severe  3.How satisfied are you with your bowel habbit?  0%  Very happy  50%  100%  Very unhappy  4. Please indicate with a cross line below how much your irritable bowel syndrome affecting or interfering with your life in general?  0%  Not at all  50%  100%  Completely |
| --- | --- | --- | --- | --- | --- | --- | --- | --- | --- | --- | --- | --- | --- | --- | --- | --- | --- | --- | --- | --- | --- | --- | --- | --- | --- | --- | --- | --- | --- | --- | --- | --- | --- | --- | --- | --- | --- | --- | --- | --- | --- | --- | --- | --- | --- | --- | --- | --- | --- | --- | --- | --- | --- | --- | --- | --- | --- | --- | --- | --- | --- | --- | --- | --- | --- | --- | --- | --- | --- | --- | --- | --- | --- | --- | --- | --- | --- | --- | --- | --- | --- | --- | --- | --- | --- | --- | --- | --- | --- | --- | --- | --- | --- | --- | --- | --- | --- | --- | --- | --- | --- | --- | --- | --- | --- | --- | --- | --- | --- | --- | --- | --- | --- | --- | --- | --- | --- | --- | --- | --- | --- | --- | --- | --- | --- | --- | --- | --- | --- | --- | --- | --- | --- | --- | --- | --- | --- | --- | --- | --- | --- | --- | --- | --- | --- | --- | --- | --- | --- | --- | --- | --- | --- | --- | --- | --- | --- | --- | --- | --- | --- | --- | --- | --- | --- | --- | --- | --- | --- | --- | --- | --- | --- | --- | --- | --- | --- | --- | --- | --- | --- | --- | --- | --- | --- | --- | --- | --- | --- | --- | --- | --- | --- | --- | --- | --- | --- | --- | --- | --- | --- | --- | --- | --- | --- |
